# Lights up on the embryonic dance: tools and applications of optogenetics in developmental biology

**DOI:** 10.1101/gad.353459.125

**Published:** 2026-05-01

**Authors:** Yang Gao, Emily K. Ho, Jared E. Toettcher

**Affiliations:** 1 Department of Molecular Biology, Princeton University, Princeton, New Jersey 08544, USA;; 2 Kravis Department of Integrated Sciences, Claremont McKenna College, Claremont, California 91711, USA;; 3 Omenn-Darling Bioengineering Institute, Princeton University, Princeton, New Jersey 08544, USA

**Keywords:** cell engineering, developmental biology, optogenetics

## Abstract

In developmental biology, cellular events must be orchestrated at precise times and locations in the embryo. Many classic discoveries were achieved by perturbing developmental organization using approaches ranging from tissue transplantation to local, acute heat shock. A growing suite of optogenetic tools is now available with fine spatiotemporal control, opening the door to perturbation experiments with unprecedented precision. Here we highlight these tools, review their application in developmental contexts, and discuss their current challenges and future promise.

## Abstract (precis)

In this review, Gao et al. discuss the recent advances in optogenetics that allow researchers to perform precise, spatiotemporally controlled, mechanistic analyses in developmental biology. While they elaborate on how optogenetic tools have been applied to dissecting developmental cellular processes and dynamics, they also articulate the challenges and future directions for the field.

What unifies developmental biologists, a group of scientists who work across diverse processes and model organisms? One link across all these contexts is that developmental systems “dance”—with groups of cells undergoing morphological movements, complex spatial patterning, and carefully choreographed transitions at specific developmental times. Different researchers may focus on different dances (tissue patterning, gastrulation, organogenesis, or regeneration) or different components of the dance (the biomechanics of the dancer, control over the tempo, or cues between dancers), but in each case, the researcher must still operate in the context of a moving collective of cells that moves to its own beat.

Studying the intrinsic spatial and temporal complexity of developmental biology's dances has always required sophisticated perturbation experiments. Transplantation of material (cells and cytoplasm) was a classic perturbation of the dance—moving dancers from one position or developmental time period to another (Spemann and Mangold 1924; Frohnhöfer and Nüsslein-Volhard 1986). As more molecular components were discovered and molecular biology advanced, perturbation experiments grew to include temperature-sensitive alleles (Suzuki 1970), the Gal4/UAS and Cre/Lox systems to drive tissue-specific gene expression (Orban et al. 1992; Brand and Perrimon 1993), or ligand-soaked beads to apply stimuli at specific locations (Yang et al. 1997). However, even these advances in spatial and temporal perturbations have substantial limitations. Spatial control is often limited by the lack of a promoter with the specific expression pattern of interest, and the pace of temporal control is generally limited by the rate of gene expression (tens of minutes to hours). Pharmacological perturbations, if available for a protein or process of interest, act quickly but often bind tightly and thus cannot be rapidly removed or localized to a specific spatial position.

What would an ideal developmental perturbation look like? Key attributes would include the ability to rapidly apply and remove the perturbation, focus it tightly in space, and be biochemically specific, activating only a single desired change in cellular activity. These key attributes are increasingly met by optogenetic tools: proteins whose activity can be controlled by the application of specific wavelengths of light. Light can be easily switched on and off for rapid stimulation, can be focused precisely in space, and in most nonphotosynthetic biological contexts is highly specific to a small set of light-sensitive proteins. A growing toolbox of light-sensitive proteins complements these strengths, achieving rapid and reversible control over many intracellular processes.

We and others have previously written reviews detailing the optogenetic toolbox and its capabilities for developmental perturbations (Repina et al. 2017; Johnson and Toettcher 2018; Goglia and Toettcher 2019; Krueger et al. 2019; Farahani et al. 2021b; Fan et al. 2022; McNamara et al. 2023). However, in the last 5 years, optogenetics has begun to realize its full potential as a broadly applicable tool for developmental biologists working across model systems and questions. This increase has been driven by design advances in optogenetic strategies that make systems more reliable and applicable in more organisms, conceptual advances in optogenetics for not only perturbing developmental systems but also directing applications such as stem cell differentiation, and technical advances in microscopy that make complex, large-scale, and dynamic optogenetic experiments feasible. Therefore, the purpose of this review is to survey this recent explosion of optogenetic applications in developmental contexts. We aim to contextualize these advances so that the developmental biologist can confidently apply this powerful technique to their application of interest. For the synthetic biologist, we also aim to articulate new frontiers in developmental biology in which the next generation of advances in optogenetic capabilities would make a significant impact.

## An introduction to optogenetics for the developmental biologist

The term “optogenetics” was first coined by Deisseroth et al. (2006) to describe a powerful new tool: genetically encoded light-sensitive ion channels for controlling neural activity. However, two light-controlled synthetic protein systems had already been introduced by 2002: a light-activated G protein-coupled receptor in *Xenopus* oocytes and mammalian neurons (Zemelman et al. 2002) and a synthetic split transcription factor for light-induced gene expression in budding yeast (Shimizu-Sato et al. 2002). In this sense, optogenetics was founded to include diverse target proteins and cellular contexts. Light control in neuroscience proceeded rapidly and gained wide adoption owing to the remarkable utility of a core set of tools—light-gated ion channels—for controlling neuron excitation in virtually any context. However, for much of cell biology, light-based control of each cellular process has been slower, requiring the development of a customized optogenetic tool for each target protein and process of interest.

Comprehensive reviews as well as the OptoBase database (https://www.optobase.org) detail the photochemistry of light-sensitive domains and fully catalog the existing optogenetic toolbox; we point interested readers to these resources (Repina et al. 2017; Krueger et al. 2019; Fan et al. 2022). In this section, our aim is to briefly introduce the practical considerations that are relevant to those considering optogenetics for the first time: What are the most commonly used light-sensitive domains in developmental biology applications, and how does one go about performing an optogenetic experiment? These principles are also necessary to contextualize the studies discussed in the remainder of the review as well as the future challenges.

### A brief overview of the optogenetic protein toolbox

Optogenetic tools are typically built from two components: a light-sensitive protein domain and a small molecule chromophore that absorbs photon energy and converts it to a chemical change (e.g., bond formation) within the protein, leading to a light-induced conformational change. This change can be exploited to drive a number of processes including protein–protein dimerization, oligomerization, dissociation/cleavage, or exposure of a peptide sequence that can be functionalized in various ways.

There are three major classes of naturally occurring light-sensitive protein domains that form the basis of the most commonly used optogenetic tools. Key distinguishing features of these light-sensitive domains are as follows: (1) What wavelength of light does the optogenetic tool respond to? The answer to this question determines both which wavelength of light is needed for stimulation and which fluorescent proteins are compatible with the optogenetic system (e.g., the 488 nm laser often used to excite GFP will also activate blue-light optogenetic systems). (2) Is an endogenous chromophore for the light-sensitive domain present in the cells, or does one need to be added? (3) What conformational change is induced in the light state, and how can it be used to impact cellular processes?

The first major family of light-sensitive domains exploit light–oxygen–voltage (LOV) domains from plants or fungi, which respond to blue light (440–473 nm) and bind flavin mononucleotide chromophores that are widely available in both prokaryotic and eukaryotic cells. Two commonly used LOV domains have different conformational changes in response to light, resulting in their use in different optogenetic applications. First, AsLOV2 from the oat plant *Avena sativa* uncages a natural effector domain that can be replaced with a functional domain of interest (Harper et al. 2003; Repina et al. 2017). For example, the improved light-induced dimer (iLID) tool uncages an *Escherichia coli* peptide, SsrA, which is then free to bind its cognate SspB binding domain (Guntas et al. 2015). This interaction was optimized to form the iLID/SspB light-induced dimerization system. Second, Vivid (VVD), the smallest known LOV-containing protein, forms a rapidly exchanging dimer upon blue-light activation, and this dimerization can be harnessed for purposes like light-induced transcriptional activation (Zoltowski et al. 2007; Zoltowski and Crane 2008; Wang et al. 2012).

The second widely used light-sensitive domain is the photolyase homology region (PHR) of *Arabidopsis thaliana* cryptochrome 2 (CRY2). Similar to LOV, the CRY2^PHR^ domain is blue-light-sensitive and binds a flavin chromophore widely available in the cell: flavin adenine dinucleotide. The conformational change in CRY2 induced by blue light can be harnessed for both light-induced dimerization and oligomerization. Under blue light, CRY2 will oligomerize or dimerize with either the cryptochrome-interacting basic helix–loop–helix (CIB1) or a truncated version (CIBN) (Kennedy et al. 2010; Bugaj et al. 2013). CRY2^PHR^ variants have been developed that favor either dimerization or oligomerization to further separate these functionalities (Taslimi et al. 2014; Duan et al. 2017).

Finally, the third major class of optogenetic tools is based on phytochrome protein domains derived from plants, photosynthetic cyanobacteria, or soil-dwelling bacteria (Tang et al. 2021). Different phytochromes can respond to diverse wavelengths of light across the entire visible and near-infrared spectrum (Rockwell et al. 2011) but are often highly sensitive to red and near-infrared (NIR) light, making them excellent tools for in vivo penetration (Tischer and Weiner 2014; Krueger et al. 2019; Lu et al. 2020). Different members of the phytochrome family bind to various related chromophores, ranging from the widely available biliverdin to the species-restricted phycobilins, so usage of a phytochrome-based optogenetic tool often involves chromophore supplementation to enable light sensitivity. The wide tissue availability of biliverdin makes biliverdin-sensitive phytochromes particularly useful for in vivo applications where chromophore delivery may be challenging (Bhoo et al. 2001; Redchuk et al. 2017; Tang et al. 2021; Kuwasaki et al. 2022). Diverse phytochrome-based optogenetic tools have already been developed for driving protein dimerization, subcellular localization changes, and gene expression (Shimizu-Sato et al. 2002; Levskaya et al. 2009; Yousefi et al. 2019; Kuwasaki et al. 2022; Böhm et al. 2025), though few have been tested so far in developmental contexts.

### Strategies for applying light to developing systems

The first question many developmental biologists embarking on an optogenetic experiment encounter is how much light is needed to activate an optogenetic tool. Optogenetic tool light intensity is typically measured in the power density per unit area (e.g., milliwatts per square centimeter). LOV and Cry2 domain-based systems are typically stimulated in the 1–10 mW/cm^2^ range, comparable with bright room light but orders of magnitude dimmer than a laser for subcellular fluorescent protein imaging, whereas phytochrome-based tools can be potently activated by even lower light intensities (Pathak et al. 2014). In most cases, the goal of illumination is to saturate the optogenetic tool in the active state, and thus precise calibration of light intensity is not necessary. However, limiting light intensity can have the advantage of reducing the phototoxicity and yielding more precise spatial patterning. In addition, the response to an optogenetic input will depend on both the stimulation intensity and the expression level of the construct, which in many cases can be variable between embryos or cells. Calibrating expression levels of optogenetic tools between embryos is one strategy that can be used to improve reproducibility (Ho et al. 2023).

**Figure 1.**
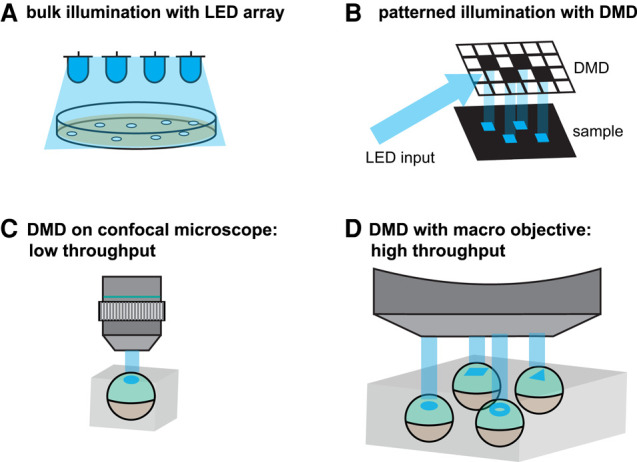
Strategies for optogenetic stimulation. (*A*) LED arrays are useful for bulk stimulation but lack the ability to apply spatial patterns. (*B*) Digital micromirror devices (DMDs) allow user-defined masks to be projected onto a sample on a confocal microscope. (*C*) Microscope-based spatial illumination typically exhibits restricted throughput because the stage must be rapidly cycled through each stimulation position to maintain optogenetic tool activity. (*D*) An alternative for high-throughput patterning is to use a DMD with a macro-objective lens that can project patterns on the millimeter to centimeter scale to simultaneously stimulate many biological samples.

With these considerations in mind, there are several strategies for illuminating embryos. The simplest approach is to use light-emitting diode (LED) arrays arranged on boards to provide light over a large surface area (Fig. 1A). These arrays can be quite simple, consisting of nothing more than standard 5 mm red–green–blue LEDs connected in series with a resistor and driven by a DC power supply, which can be assembled for $100 or less. More sophisticated designs can incorporate microcontrollers to tune LED intensity and the frequency of illumination, for example, in different wells of a multiwell plate, and both open source and commercial designs are now readily available (Hennemann et al. 2018; Bugaj and Lim 2019; Repina et al. 2020; Höhener et al. 2022). These approaches are most useful for bulk stimulation of a sample; for instance, many embryos in the same dish.

How might one apply spatial perturbations on the length scale of a developing tissue or embryo? Two simple options are immediately available to most developmental biologists. One is to use the intrinsic capability of point-scanning confocal microscopes to deliver localized illumination patterns, though the stimulus intensity of a point-scanning laser source is typically orders of magnitude greater than that needed for optogenetic tool activation and can lead to considerable activation outside of the desired region. Another straightforward option is to place a pattern mask in the light path. The mask can be placed on a microscope condenser to pattern light from a transmitted light source or placed on a sample plate illuminated by an LED array (Repina et al. 2020; Suh et al. 2025). However, this approach does not permit the pattern to be altered except by fabricating a new mask.

A third widely used approach is to employ a digital micromirror device (DMD), a 2D array of mirrors where each mirror can be treated as a stimulation pixel, redirecting light to the tissue in a spatial pattern (Fig. 1B). Typically, a DMD can be placed in between a light source and objective lens on a microscope to focus the illumination pattern within the imaging field of view (Fig. 1C). The pixel size scales with the objective magnification (a typical commercial DMD pixel size is 10 µm divided by the objective magnification) so that a lower-magnification objective also produces coarser illumination patterns.

The primary drawback of all three approaches discussed above is the relatively low throughput of microscope-based optogenetic stimulation for developmental samples. An automated XY stage must cycle between samples rapidly enough that optogenetic stimulation is maintained between cycles. For many developmental applications, it may be preferable to combine a low-magnification macro-objective lens, DMD, and light source in a low-cost patterned illumination system to simultaneously illuminate an ∼1 cm^2^ area containing many biological samples (Fig. 1D; McNamara et al. 2025). Higher-throughput stimulation does present a trade-off, as the same number of DMD pixels are used to illuminate a broader area, limiting the spatial resolution of the applied pattern.

## Optogenetic control of the developmental cell

Developmental biologists often conceptually group processes as being involved in either cell signaling, gene expression, or morphogenesis. Similarly, we encourage the developmental biologist to make sense of an ever-growing optogenetic toolbox by grouping tools in these same categories. Although there are many strategies for accomplishing a task such as turning on a signaling pathway with light, the possibilities and limitations are similar no matter which light-sensitive domain is used.

The goal of this section is to review key optogenetic strategies that have already been successfully applied to multicellular contexts and highlight the common challenges facing each approach. Our examples are drawn from a multitude of classic developmental contexts: *Drosophila*, *Xenopus*, zebrafish, and mammalian embryo models.

### Controlling developmental signaling: receptor activation

One broadly successful strategy for controlling developmental signal transduction has been to induce receptor activity. A benefit of activating pathways at the receptor level is that many receptors simultaneously activate multiple downstream effectors, and thus optogenetic receptors can fully recapitulate the effects of ligand binding.

**Figure 2.**
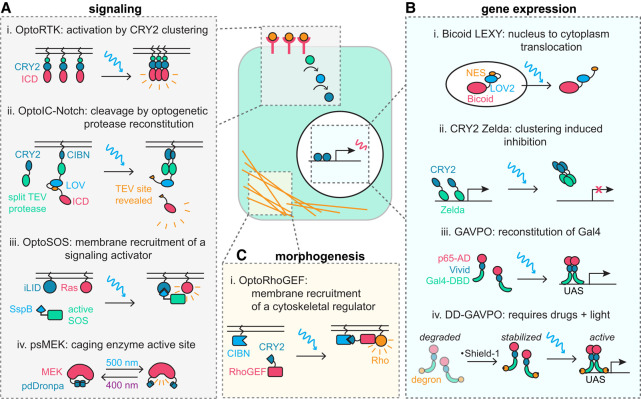
The optogenetic cell. The optogenetic toolbox contains tools for manipulating key developmental processes including signaling (*A*), gene expression (*B*), and morphogenesis (*C*). (*A*) Signaling can be controlled at the receptor or downstream effector level. (Panel *i*) OptoRTKs cluster the intracellular domains (ICDs) of receptor tyrosine kinases using CRY2-based clustering. (Panel *ii*) OptoIC–Notch uses CRY2/CIBN dimerization to reconstitute a split TEV protease and a LOV domain to reveal a TEV cleavage site, resulting in release of the Notch intracellular domain (ICD) upon blue-light stimulation. (Panel *iii*) OptoSOS uses a membrane-tethered iLID to recruit the catalytic domain of SOS fused with SspB to the membrane. (Panel *iv*) In psMEK, the active site is revealed by dissociation of pdDronpa domains. (*B*) Gene expression can be controlled through optogenetic manipulation of developmental transcription factors or Gal4. (Panel *i*) Fusion of LEXY to transcription factors results in light-induced nuclear export due to the reveal of a nuclear export sequence (NES). (Panel *ii*) Fusion of CRY2 to transcription factors can result in inhibition due to clustering. (Panel *iii*) GAVPO is a light-inducible version of Gal4 that uses the Vivid domains to dimerize subunits containing the Gal4 DNA binding domain (Gal4-DBD) and the p65 activation domain (p65-AD). (Panel *iv*) An updated DD-GAVPO adds drug-inducible stabilization of the transcription factor using Shield-1 for greater control over the off state. (*C*) Cell morphogenesis can be controlled through optogenetic manipulation of the cytoskeleton. An OptoRhoGEF uses CRY2–CIBN dimerization to bring a RhoGEF to the membrane, where it activates Rho.

Optogenetic tools induce receptor activity by mimicking the natural response to ligand binding. Several classes of receptors dimerize or cluster upon ligand binding, including receptor tyrosine kinases (RTKs), bone morphogenetic protein (BMP) receptors, and Nodal pathway activin receptors, leading to pathway activation. Ligand-independent receptor activation can be achieved by fusing the receptor's intracellular-facing sequence to an optogenetic tool that triggers light-inducible homodimerization (e.g., VVD) (Grusch et al. 2014; Sako et al. 2016; Rogers et al. 2020) or clustering (e.g., CRY2) (Fig. 2A, panel i; Bugaj et al. 2013; Farahani et al. 2021a; Yadav et al. 2021; Iannucci et al. 2025). This approach has been successful in both *Drosophila* and zebrafish embryos.

A challenge for ectopic expression of light-induced receptors is that in many cases, overexpression is a well-established mechanism for ligand-independent activation (Du and Lovly 2018). Overexpression of optogenetic receptors can have the same effect. The use of optogenetic receptors can thus be limited by either dark state receptor activation or, if leaky receptor activity results in pathway adaptation, weak inducibility. Therefore, it is critical to choose receptor expression levels that produce a strong separation between dark state- and light-induced activity. Krishnamurthy et al. (2020) developed an alternative strategy to reduce dark state activation of an optogenetic receptor using light-controlled fibroblast growth factor receptor (FGFR) in *Xenopus* embryos as a model. They separated the membrane anchor from the cytoplasmic signaling domain and fused them to CIBN and CRY2^PHR^, respectively. Light stimulation of CRY2 induces both heterodimerization to CIBN and CRY2 clustering so that two activation events—membrane recruitment and receptor clustering—are controlled by light. A similar strategy was also successfully applied to Nodal receptor activation in zebrafish (McNamara et al. 2025), suggesting that it might be of general utility.

Receptors activated by processes other than oligomerization have also been placed under optogenetic control. For example, the Notch receptor is activated via cleavage of the Notch intracellular domain. To mimic this natural process in *Drosophila* embryos, an optogenetic version of the Notch receptor uses light to both reveal a TEV protease cleavage site in and reconstitute a TEV protease at the receptor, resulting in blue-light-activated cleavage (Fig. 2A, panel ii; Townson et al. 2023). As described previously for receptor membrane translocation and clustering, controlling two biochemical events with light leads to lower leakiness and higher dynamic range compared with controlling a single event.

G protein-coupled receptors (GPCRs) have also been placed under optogenetic control through the assembly of chimeras that contain intracellular domains from a GPCR of interest with transmembrane domains from the light-sensitive rhodopsin GPCR (Airan et al. 2009). This strategy was applied recently to noncanonical Wnt signaling to produce a light-controlled variant of the Frizzled receptor in zebrafish embryos (Čapek et al. 2019).

A notable exception in this list of successfully light-controlled receptors is that of Patched, the receptor in the Sonic hedgehog pathway. The mechanistic picture of how Patched is activated has improved in recent years (Zhang and Beachy 2023), suggesting that this receptor may be ripe for optogenetic control.

### Controlling developmental signaling: signal transduction

In addition to activating signaling at the receptor level, optogenetics can be used to regulate many aspects of the intracellular signal transduction machinery. The ability to “walk down” a pathway and apply stimuli at different nodes is of particular interest for researchers seeking to identify sources of amplification or feedback regulation or in cases where it is desirable to activate just a single pathway downstream from a receptor with multiple outputs. The Ras/ERK mitogen-activated protein kinase cascade downstream from receptor tyrosine kinases exemplifies this kind of control, as multiple tools are now available to control the pathway at different nodes. Briefly, Ras is a small G protein whose GTP loading triggers activation of the Raf kinase. Active Raf phosphorylates its substrate MEK, which in turn phosphorylates ERK to regulate diverse substrates including transcription factors to trigger a variety of cellular responses. Here we describe optogenetic tools to activate Ras and MEK as representative strategies for controlling signal transduction but note that tools also exist for optogenetic control of Raf that have been applied in *Xenopus* and *Drosophila* (Aoki et al. 2013; Wend et al. 2014; Zhang et al. 2014; Krishnamurthy et al. 2016; Wang et al. 2020).

Many cellular events, including signal transduction, are mediated by recruitment of an effector from the cytoplasm to the plasma membrane. Therefore, a useful and generalizable optogenetic strategy is to take advantage of optical dimerization systems to trigger light-induced membrane recruitment, such as the iLID–SspB dimerization system (Guntas et al. 2015). By tethering iLID to the plasma membrane and expressing SspB-fused cargo in the cytosol, the cargo can be recruited to the membrane upon blue-light stimulation. The OptoSOS system for Ras activation uses this principle to recruit SOScat, the catalytic domain of the guanine nucleotide exchange factor (GEF) Son of Sevenless (SOS), to the membrane and activate Ras (Fig. 2A, panel iii; Toettcher et al. 2013; Johnson et al. 2017). In *Drosophila*, OptoSOS has been used extensively to study input/output relationships between Ras/ERK pathway activity and gene expression (Johnson and Toettcher 2019; Johnson et al. 2020; McFann et al. 2021; Ho et al. 2023).

Another strategy for regulating signaling enzymes is to take advantage of the feature that many signaling enzymes are autoinhibited by intramolecular association between domains to cage an active site (Yeh et al. 2007). Zhou et al. (2017) demonstrated that flanking a constitutively active kinase domain with photoswitchable dimerization domains (pdDronpa monomers) and appropriate linkers can achieve the same molecular architecture. The approach was applied to cage an otherwise constitutively active MEK mutant to generate photoswitchable MEK (psMEK) (Fig. 2A, panel iv). Adding additional activating mutations produced an optimized psMEK for use in *Drosophila* and zebrafish and has proven useful for directly studying the effects of ERK activation/inhibition on its target, Capicua, a transcriptional repressor of developmental patterning genes (Patel et al. 2019, 2021).

A theme uniting the OptoSOS and psMEK systems is that both tools start with constitutively active versions of signal effectors and then restrict their activity through either mislocalization or active site caging in a manner that can be rescued with light. The advantage of this approach is that expression of the optogenetic tool is not expected to substantially interfere with endogenous signaling in the dark (because the engineered protein's activity is restricted), and light-induced activity cannot be altered or reduced by endogenous regulatory inputs (because the core signaling protein is constitutively active).

### Controlling transcription factor activity

Controlling the activity of endogenous transcription factors has broad use for developmental biologists, both to study the mechanisms of gene regulation and to directly induce signaling or cell fate states in a cell population of interest. Strategies for optogenetic control over transcription factor activity are based primarily on the principle of allowing or restricting transcription factor access to DNA in the nucleus.

One strategy for controlling the activity of a transcription factor is to control its nuclear localization by fusing it to LEXY (light-inducible nuclear export system), LINuS (light-inducible nuclear localization sequence), or LANS (light-activated nuclear shuttle) (Niopek et al. 2014, 2016; Yumerefendi et al. 2015). These tools use AsLOV2, which undergoes a conformational change upon blue-light stimulation, to reveal either a nuclear export signal or a nuclear localization signal. For example, the *Drosophila* transcription factor Bicoid has been fused to LEXY, and the Hippo pathway transcription factor YAP has been fused to both LEXY and LINuS in zebrafish and stem cell models (Fig. 2B, panel i; Singh et al. 2022; Toh et al. 2022; Meyer et al. 2023). The greatest challenge facing successful application of these tools is achieving complete translocation out of the nucleus in either the lit state (for LEXY) or dark state (for LINuS/LANS); as a result, these tools typically exhibit some leaky gene expression in the off state. An improved LEXY (iLEXY) showed a reduction in this leakiness in *Drosophila* embryos at the expense of slower kinetics by incorporating mutations that slow down the protein's cycling back to the dark state (Kögler et al. 2021), though why leaky nuclear localization is reduced under continuous, saturating illumination remains unclear.

There are other strategies for controlling developmental transcription factor activity. One is to introduce transcription factors fused to the oligomerizing CRY2^PHR^ domain. Light-induced oligomerization of CRY2–transcription factor fusions leads to inhibition of some transcription factors. This is the case for Bicoid as well as the *Drosophila* pioneer factor Zelda (Fig. 2B, panel ii; Huang et al. 2017; McDaniel et al. 2019). However, the effects of transcription factor clustering can be diverse: Transcriptional hubs or condensates are thought to be necessary for some transcription factors to function (Boija et al. 2018; Du et al. 2024; Munshi et al. 2025), and in some cases, light-induced oligomerization of transcription factors has been shown to enhance gene expression (Schneider et al. 2021; Brumbaugh-Reed et al. 2024; Yang et al. 2024a). Thus, CRY2 clustering is not a universal method for transcription factor control.

Another strategy is to use AsLOV2 to expose a protein destabilization sequence upon blue-light stimulation (Irizarry et al. 2020). This optogenetic domain is known as a “blue-light-inducible degron” (BLID) (Bonger et al. 2014). Compared with LEXY or CRY2, BLID is not reversible on fast timescales due to the requirement for new protein synthesis to restore the unstimulated state. However, its effect may also be more potent. One study in *Drosophila* compared the consequences of optogenetic inhibition of the Dorsal transcription factor using LEXY and BLID and found that each strategy had different effects on transcription of the target gene *snail*, likely attributable to continued low-level nuclear import and export of Dorsal-LEXY even in the illuminated state where export is favored (McGehee and Stathopoulos 2024). This study highlights the importance of considering the details of one's optogenetic tools of choice, as these subtleties can impact developmental processes.

### Controlling gene expression

For many applications, it would be desirable to apply light as an inducer of gene expression. However, building useful optogenetic tools for light-inducible gene expression in embryos has proven to be challenging to implement. The primary obstacle for optogenetic control of transcription is that, similar to systems previously discussed, the dark state must be transcriptionally silent, but the light state must achieve rapid and robust gene expression.

One strategy has been to use a light-inducible Gal4 protein that can be paired with a UAS-driven gene of interest. GAVPO is a light-activated Gal4 that contains a DNA binding domain, a Vivid (VVD) photosensitive dimerization domain, and a p65 transcriptional activation domain (Fig. 2B, panel iii; Wang et al. 2012). GAVPO dimerizes under blue light to activate transcription at UAS promoters. However, although GAVPO works well in cultured cells and in vivo in mice, it proved toxic in zebrafish embryos and has not been used successfully in *Drosophila* (Reade et al. 2017).

Despite the abundance of other optogenetic tools in *Drosophila*, light-based control of gene expression long remained unavailable. Di Pietro et al. (2021) sought to address this limitation by building ShineGal4, which uses the heterodimerizing pair of domains in the Magnet light-induced heterodimerization system to bring the DNA binding and transcriptional activation domains of Gal4 together upon blue-light stimulation. ShineGal4 robustly induces gene expression in *Drosophila*, achieving a 30-fold increase in gene expression from a low initial level. It is still unclear precisely how rapidly transcription is induced by ShineGal4, as measurements to date have been limited to fluorescent protein accumulation, which combines the timescales of transcription, protein synthesis, and fluorophore maturation.

Use of GAVPO is also limited by its residual dark state activity, which has been demonstrated in multiple developmental systems (Benzinger and Briscoe 2025; Varady et al. 2025). To address this challenge in mouse neural progenitors, Benzinger and Briscoe (2025) added a small molecule (Shield-1)-responsive conditional degron domain to GAVPO (DD-GAVPO) so that expression required both light and small molecule addition (Fig. 2B, panel iv). This system provides exceptional control over gene expression because basal expression is low, inducibility is robust, expression can be tuned with either light amplitude or Shield-1 concentration, and induction of gene expression is reversible. Other studies have opted to use an optogenetic Cre/Lox system in which a split Cre recombinase is reconstituted by light-induced dimerization domains. The optogenetic Cre system has been shown to have low dark state activation in zebrafish embryos and human embryonic stem cells, and the off state can be further enforced by putting Cre expression under control of a drug-inducible or heat shock-inducible promoter (De Santis et al. 2021; Varady et al. 2025). For example, a recent zebrafish tool called zHORSE (zebrafish for heat shock-inducible optogenetic recombinase expression) uses a heat shock promoter to drive expression of a split Cre recombinase that reconstitutes under blue light via VVD dimerization domains (Varady et al. 2025). zHORSE had much less dark activity compared with GAVPO in zebrafish, suggesting that this dual gating of optogenetic tools for gene expression may be a generalizable strategy.

Other possibilities for modulating gene expression with light include photoprotected mRNAs, photocaged morpholinos, and inducible mRNA degradation (Love et al. 2025; Tarbashevich et al. 2025; Weissenboeck et al. 2025). These systems promise more rapid control over gene expression than is possible with transcriptional induction. However, additional work is necessary to make these systems fully transgenic or to allow them to target specific genes of interest.

### Controlling the cytoskeleton

Cytoskeletal perturbations have been at the forefront of optogenetic tool development since the field's inception (Levskaya et al. 2009; Wu et al. 2009) due to the importance of acute and localized subcellular perturbation for manipulating cell migration programs. The cytoskeleton is a central player in developmental systems, contributing to processes such as migration, tissue morphogenesis, and polarized cell division. Light-controlled cytoskeletal manipulations were thus among the first optogenetic applications in developmental biology.

A pioneering study by Wang et al. (2010) used a photoactivatable form of the small GTPase Rac to dissect its role in collective border cell migration in *Drosophila* egg chambers. Photoactivatable Rac (PA-Rac) fuses a constitutively active Rac to AsLOV2. In its dark, closed conformation, AsLOV2 blocks Rac's interactions with its effectors. Upon light stimulation, the conformational change in AsLOV2 releases the steric hindrance, allowing Rac activation (Wu et al. 2009). PA-Rac has been used to direct migration of *Drosophila* border cells as well as zebrafish neutrophils in vivo (Wang et al. 2010; Yoo et al. 2010).

Subsequent studies expanded the toolbox for light-based control of the cytoskeleton by developing optogenetic tools that recruit other constitutively active G protein regulators or lipid-modifying enzymes to the plasma membrane. Guglielmi et al. (2015) developed a tool to deplete actin from the *Drosophila* cell cortex by using the CRY2–CIBN dimerization pair to bring the catalytic domain of a PI(4,5)P_2_ phosphatase to the membrane. Similar recruitment-based approaches were used to modulate Rho activity and downstream myosin contractility in *Drosophila* by localizing a Rho GEF (RhoGEF2), a Rho GAP (Gap43), and a dominant negative version of Rho to the membrane (Fig. 2C; Izquierdo et al. 2018; Rich et al. 2020; Herrera-Perez et al. 2021; Guo et al. 2022). More recently, similar approaches have been extended to modulating the localization of endogenous *Drosophila* Rho GEFs (Countryman et al. 2025).

One challenge with using this set of tools is that they require tightly localized subcellular stimulation to produce asymmetric contraction at a particular tissue interface (e.g., apical constriction and tissue invagination), typically achieved using two photon excitation of the optogenetic system at a particular membrane surface of interest. To address this limitation, Martínez-Ara et al. (2022) developed OptoShroom3, a split variant of the Shroom3 regulatory protein whose function is reconstituted by light-induced dimerization. The N-terminal actin-binding domain confers apical localization, whereas the C-terminal ROCK interaction domain recruits Rho kinase to regulate contractility. In this manner, both the location and activity are genetically encoded, and global illumination was shown to be sufficient to reconstitute apical contractile responses in mouse and human neural organoids as well as in cultured cell models. It might be the case that motifs like the N-terminal localization domain of Shroom3 could be combined with a broader array of functional domains to induce subcellular responses under simple illumination patterns.

## Illuminating development with temporal and spatial control

Having described the major classes of optogenetic tools that are immediately available to the developmental biologist, we now turn to a discussion of the experimental strategies that harness their unique capabilities to generate developmental insights. We highlight how high temporal and spatial precision makes these experiments possible and also consider areas where optogenetics holds promise for key open questions.

### Temporal control

Optogenetics offers two distinct temporal advantages compared with other approaches for manipulating cellular processes. First, many light-sensitive proteins switch conformation rapidly upon the application and removal of light stimuli, providing the opportunity for fast and reversible manipulation of cellular states. Although pharmacological manipulations, if available, can also act rapidly and reversibly, cycling between on and off states requires a laborious and thorough washout of drugs, which itself can perturb cellular processes. Second, time-varying inputs can be combined with other forms of control (e.g., focusing on particular spatial regions or varying light intensity to precisely control signal amplitude) to generate even more complex and dynamic patterns than are possible with drugs or other modes of genetic manipulations. Here we focus on how these advantages have been leveraged for new insights.

#### Defining the kinetics of developmental processes

**Figure 3.**
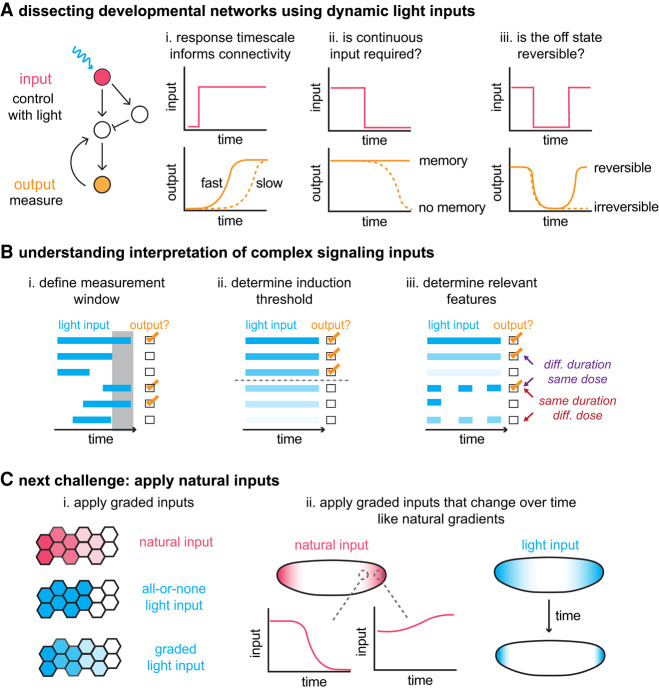
Dissection of genetic networks and signal interpretation mechanisms with dynamic light inputs. (*A*) Pairing optogenetic inputs with live output measurements has allowed kinetic questions about genetic networks to be addressed. (Panel *i*) Measuring the response timescale to an input can reveal whether network interactions are direct or indirect. (Panels *ii*,*iii*) Inputs can also be switched off and then back on to assess the memory and reversibility of an output state. (*B*) To understand signal interpretation, optogenetics allows the researcher to manipulate multiple features of an input including both the duration and amplitude. (Panel *i*) By applying light inputs over different windows, it is possible to define the measurement window of the system. (Panel *ii*) By applying light of different intensities, it is possible to define the threshold to induce an output of interest. (Panel *iii*) By simultaneously and systematically manipulating both duration and amplitude, it is possible to determine whether outputs depend on input amplitude, duration, or integrated dose. (*C*) The next challenge for dissecting these processes is to apply more natural inputs, including graded inputs in space (panel *i*) and temporally evolving patterns that mirror the dynamics of natural patterning (panel *ii*).

Developmental gene networks, where nodes are transcription factors and edges are gene expression relationships, provide illustrative examples where dynamic light inputs were used to uncover new relationships (Fig. 3A). Classical genetics and epistasis experiments help us to order the events in a developmental network and determine the necessity and sufficiency of each node. However, because genetic perturbations are permanent, these approaches often fail to address the temporal relationships between nodes, which are often critical for understanding the mechanism. By simply turning on and off inputs with light-based control and monitoring the responses of other nodes, it is possible to derive critical insights about network connectivity and kinetics. Optogenetics has been particularly well suited for studying the model genetic networks in *Drosophila* embryos, where any drug manipulations require injections and thus lack reversibility.

Even for well-studied nodes in a protein or gene network, it can be unclear whether they act directly or indirectly on downstream processes. By acutely controlling activity at a node with light, one can measure the timescale of downstream events to infer whether interactions are direct (Fig. 3A, panel i). Using these principles, Singh et al. (2022) studied the Bicoid transcription factor, which is thought to function as an activator, directly activating some gap genes and indirectly repressing others. They used LEXY to control Bicoid localization in the nucleus and the MS2–MCP system to make real-time measurements of gap gene transcription in response to these perturbations. Unexpectedly, they found that *knirps*, which was thought to be indirectly repressed by Bicoid, responded to changes in Bicoid activity within minutes. This minutes-long timescale is consistent with direct repression, suggesting that Bicoid unexpectedly acts in a direct manner to repress the *knirps* locus.

Acutely turning off a stimulus that was previously applied can also help determine whether a given process requires continuous input or whether, once activated, it can sustain itself (Fig. 3A, panel ii). For example, are pioneer factors (transcription factors that open chromatin and enable access to other transcriptional regulators) continuously required over the duration of genome activation or only transiently necessary to set future steps of genome activation in motion? McDaniel et al. (2019) asked this question with regard to Zelda, a pioneer factor in *Drosophila*. They fused endogenous Zelda to the CRY2^PHR^ domain, which clusters upon blue-light stimulation and inhibits Zelda function. They then applied blue light to inactivate Zelda over different temporal windows, corresponding to the major and minor waves of zygotic genome activation, and performed RNA sequencing. They found that Zelda was indeed continuously required for full genome activation. However, for some genes, especially those that are activated early, a transient pulse of early Zelda activity is sufficient.

Finally, toggling between on and off states of an input can reveal whether outputs are reversible and whether that reversibility depends on features such as the duration of the input or the number of reversals (Fig. 3A, panel iii). Zhao et al. (2024) used this approach to investigate how the *Drosophila* transcription factor Knirps functions as a repressor. They directly tested whether repression by Knirps was reversible by applying blue light to translocate LEXY-Knirps out of the nucleus after it had already established repression of *eve*. By measuring transcription of *eve* using an MS2 reporter, they found that repression was reversible, as removal of Knirps allowed *eve* transcription to resume. Importantly, they determined that transcription recovers within minutes after Knirps removal and that this recovery timescale is not dependent on the duration of repression.

All three of these examples describe efforts to understand the mechanisms of gene regulation. It is possible to imagine applying similar strategies to dissect other processes, such as signal transduction cascades, where an input at one pathway node could be tracked as it propagates through a signaling cascade. The challenge is that these experiments require sensitive measurements of downstream responses, akin to the MS2-MCP system for transcription, to pair with the acute optogenetic perturbation, and signaling biosensors are still relatively restricted to a few major nodes. In a related recent effort, Yang et al. (2024b) paired optogenetic activation of the Ras/ERK pathway with phospho-proteomics to identify phosphorylation targets of ERK in the *Drosophila* embryo. Despite the embryo being enclosed in an eggshell and vitelline membrane that prevent rapid delivery of external chemical stimuli, samples could be rapidly collected within minutes after illumination, ideally limiting responses to direct phosphorylation targets of ERK. Although the results of the study suggested that indirect targets were also phosphorylated, this study represents exciting progress toward using optogenetics to dissect rapid signaling events.

#### Dissecting signal interpretation with dynamic inputs

During development, cell movement and differentiation are controlled by local extracellular cues. However, what features of these cues are actually sensed by cells to regulate their decisions—the strength of signaling, its duration, or some complex combination of both (Fig. 3B)? A growing set of studies has begun to address this question across diverse developmental contexts using light-based control.

Several studies in *Drosophila* and zebrafish have applied light during different temporal “windows” to assess when cells are capable of initiating a response to a signaling cue (Fig. 3B, panel i; Sako et al. 2016; Huang et al. 2017; Johnson et al. 2017; Irizarry et al. 2020; McFann et al. 2021; Emig et al. 2025; Pimmett et al. 2025). It is similarly possible to manipulate signal amplitude by simply adjusting the brightness of the light. Applying light inputs with different amplitudes has been useful for assessing signaling thresholds (Fig. 3B, panel ii; Rogers et al. 2020; Yadav et al. 2021; Ho et al. 2023). It is notable that optogenetic stimuli can sometimes achieve signal amplitudes and cellular responses that cover a wider range than gain-of-function mutants (Johnson and Toettcher 2019), whether due to the acute nature of the optogenetic stimulus (avoiding long-term desensitization) or the specific node being activated (avoiding negative feedback at upstream nodes).

Optogenetics is notable for its ability to manipulate both signal timing and amplitude in vivo with a high level of precision to uncover more complex signal processing relationships between the cellular stimulus and response (Fig. 3B, panel iii). For example, Johnson and Toettcher (2019) used OptoSOS, an optogenetic tool for activating ERK signaling, to determine whether the maximum amplitude, total duration, or “area under the curve” of stimulation dictated cellular responses to ERK in *Drosophila* embryos. They found that the cumulative dose was most predictive of embryo phenotype. The same response could be induced by a long and dim input, a short and bright input, or multiple bright pulses. These studies are just a beginning, and deeper insights remain to be uncovered through developmental stimulus–response experiments. For example, one might perform “screens” of signal interpretation across a broad range of inputs (Rosen et al. 2025), test the limits of signal perception in the presence of different amounts of noise, or even infer molecular network architecture by determining how different inputs map to outputs (Mettetal et al. 2006).

Future studies must also combine the ability to vary patterns in time and space (Fig. 3C). This capability is particularly important because many developmental signaling patterns never reach a steady state. For example, both the terminal ERK gradient in *Drosophila* and the Sonic hedgehog gradient in the vertebrate neural tube evolve over time due to factors such as ligand trapping and tissue growth (Chamberlain et al. 2008; Coppey et al. 2008). Dynamic inputs are well within the current capabilities of light stimulation technology (Oatman et al. 2025; Passmore et al. 2025; Hinderline et al. 2026) but are limited by relatively poor understanding of how endogenous patterns quantitatively change over time. Here, biosensors of endogenous patterns of ligand stimulation and intracellular kinase activity have an important role to play. For example, a new biosensor for receptor tyrosine kinase activity revealed unexpected dynamics of the endogenous Torso receptor in the *Drosophila* terminal patterning system that may be required to maintain relatively constant ERK output (Ho et al. 2025), suggesting that there is still much to learn about how this classic signal transduction system is built.

### Spatial control

Every developmental cell biologist recognizes the power of tissue-specific promoters for delivering perturbations to appropriate cells at specific developmental time points. Optogenetic control has the potential to supercharge this capability by generating light-induced responses in any subpopulation of cells, unconstrained by whether that pattern can be encoded in a gene regulatory sequence. In this section, we highlight examples of recent studies that take advantage of the spatial power of optogenetics to perturb and direct developmental events, including exciting new applications of optogenetics to direct differentiation in mammalian organoids.

#### Precise perturbations in space

**Figure 4.**
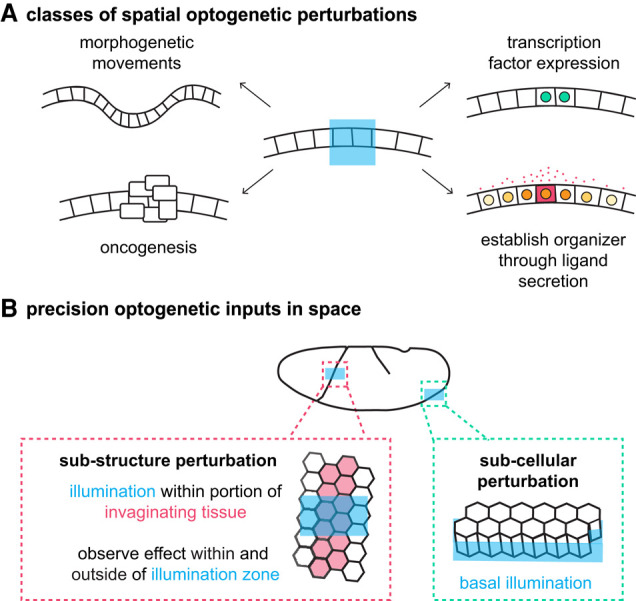
Spatial control over developing tissues. (*A*) Optogenetic tools make spatial perturbations of many forms possible. Within a region specified by illumination, cell contraction or movement can be induced to drive morphogenetic movements in tissues, developmental transcription factors can be induced to drive cell fates, oncogenic mutations can be induced, and light-inducible expression of morphogens can be used to establish organizers that pattern tissues beyond the illumination center. (*B*) The precision of optogenetics permits perturbations that are applied to a subregion of a tissue or structure of interest (such as the *Drosophila* cephalic furrow) or applied subcellularly (such as to only the basal side of an epithelium).

Optogenetics makes it possible to apply developmental inputs of many types to a user-defined region of an embryo, organoid, or other sample of interest (Fig. 4A). The user-defined region might correspond with the boundaries of a cellular population but also might target just a subportion of a tissue (e.g., inhibiting myosin contraction within only a portion of an invaginating tissue) (Eritano et al. 2020) or a subcellular domain (e.g., stabilizing myosin on just the basal side of epithelial cells) (Fig. 4B; Krueger et al. 2018). For example, a recent study used this capability to induce oncogenesis at precisely defined locations within “minicolons” grown in microfluidic scaffolds (Fig. 4A; Lorenzo-Martín et al. 2024). The investigators directed cells toward an oncogenic fate using an OptoCre system to initiate mutations in *Apc*, *Kras*, and *Trp53.* Importantly, these recombination events were induced at specific locations within the minicolon (crypt vs. luminal epithelium) to compare how tumor context influences growth and gene expression.

When an optogenetic perturbation is applied to a cell population, the biologist will naturally measure the response within those cells. However, an exciting consequence of precision spatial patterning is the ability to create boundaries between illuminated and unilluminated regions. While the illuminated cells are directly impacted by the perturbation, the neighboring, unilluminated cells will also be affected by mechanical changes in the tissue or the production of new extracellular factors. A popular use for this approach is to apply mechanical perturbations of varying strength, timing, or position and then make direct observations of individual cell shape changes or larger-scale tissue movements to infer mechanical principles (Fig. 4A; Guglielmi et al. 2015; Izquierdo et al. 2018; Krueger et al. 2018; Guo et al. 2022; Mitchell et al. 2022; Dey et al. 2025). Although techniques that induce genetic clones within a tissue (e.g., MARCM) benefit from the same principle, the high control offered by optogenetics makes new types of manipulations possible.

An elegant study by Eritano et al. (2020) illustrates how optogenetic induction of a mechanical perturbation within a subregion of a tissue that is otherwise difficult to manipulate can lead to new insights. During *Drosophila* gastrulation, the cephalic furrow is an epithelial fold that forms at a precise position along the anterior–posterior axis but does not correspond to any known gene regulatory sequence. The fold is driven by myosin II contraction along the lateral interface of a single row of cells and displays striking linearity. Eritano et al. (2020) asked whether there is mechanical coupling across the furrow to ensure linearity. Using optogenetic recruitment of a dominant negative Rho1, they inhibited myosin II contraction in a subregion of the cephalic furrow (Fig. 4B). The investigators observed that illuminated cells failed to invaginate. However, they also observed an effect in the unilluminated cells: Although these cells were still able to invaginate, they were no longer linearly aligned. These results support a model in which mechanical coupling across the cephalic furrow coordinates tissue movement.

There is growing interest in using optogenetics to induce developmental organizers: local signaling centers to orchestrate tissue-wide developmental processes. In this case, an optogenetic input induces expression of a signaling ligand in the illuminated cells and then diffuses extracellularly to create a morphogen gradient (Fig. 4A; De Santis et al. 2021, 2025; Legnini et al. 2023; Benzinger and Briscoe 2025). Importantly, the developmental biologist can precisely define the position and size of the organizer, which will impact the properties of the resulting gradient. A recent report by Benzinger and Briscoe (2025) used this principle not only to induce a Sonic hedgehog (Shh) morphogen gradient but also to study properties of morphogen transport. They used a fully reversible GAVPO-based optogenetic expression system to stimulate Shh expression in a local domain of 2D cultures of mouse neural progenitors. The investigators observed a resulting pattern of gene expression that mirrored neural tube patterning. They then made perturbations to the system—including expressing a version of Shh that lacks the C-terminal cholesterol modification and adding Scube2, an Shh interacting protein, to the extracellular media—and measured changes to the range of patterning. They also took advantage of the reversibility of this optogenetic expression system to measure the clearance rate of Shh from the extracellular space. These experiments provide important biochemical mechanistic detail about Shh morphogen transport in vivo that will inform future efforts to pattern tissues while also presenting a successful approach for optogenetic induction of an organizer.

It is exciting to envision how spatial optogenetic control can be used not only to study morphogen gradients or mechanical events but to apply precision inputs to direct differentiation in organoids. For example, Martínez-Ara et al. (2022) used OptoShroom3 to induce apical constriction in neural organoids. By applying light to a local region of the neural organoid, they induced morphological changes such as epithelial thickening, reduced lumen size, and flattened tissue (Martínez-Ara et al. 2022). Optogenetic control over gene expression has similarly been used to drive Sonic hedgehog ligand expression in a defined region of neuralizing hESCs or human neural organoids (De Santis et al. 2021; Legnini et al. 2023). We envision that optogenetics will allow the biologist to precisely apply standardized inputs in space and time and even tune these inputs to particular features of an individual organoid, improving the capabilities and reproducibility of differentiation protocols (De Santis et al. 2025).

#### The grand challenge of a developmental rescue

For any optogenetic perturbation of a developmental process, one may ask whether the perturbation “completes” the process. In other words, can the underlying biochemical signal be fully replaced by a light pattern to reconstitute normal development? Using the *Drosophila* embryo, Johnson et al. (2020) showed the first example of how an optogenetic input can fully rescue the action of a natural patterning system (Johnson et al. 2020). In the embryo, ERK signaling at the poles is necessary for development of the head, tail, and gut structures. Embryos lacking ERK signaling, such as *trunk* mutant embryos that fail to make functional ligand, are embryonic-lethal. Reasoning that optogenetic activation of the ERK pathway (OptoSOS) might be able to substitute for the endogenous ERK gradient, the investigators applied blue-light stimulation to the poles of a *trunk* mutant embryo and remarkably found that this pattern was sufficient to fully rescue development of the embryo. These rescued embryos hatched and developed into fertile adult flies.

It is notable that full rescue was obtained in only ∼30% of illuminated embryos, a viability gap with several possible causes that highlight the complexity of these experiments. Rescuing a higher percentage might be possible with improved technical control: standardization of optogenetic construct expression levels or delivering light at more precisely controlled developmental times and locations. Alternatively, the low percentage may reflect the importance of Ras-independent signaling pathways that the OptoSOS input does not activate. Future studies that better control for sources of technical variability will undoubtedly strengthen the biological conclusions about developmental robustness that can be drawn from such experiments.

Johnson et al. (2020) were able to identify a rescue condition relatively rapidly using prior genetic knowledge to determine that Ras stimulation was likely sufficient for rescue and extensive quantitative characterization of the endogenous terminal pattern to determine when and where an optogenetic input should be applied (Lu et al. 1993; Coppey et al. 2008). However, in many cases, successful rescue will require the systematic testing of many conditions and thus the technology to pattern many embryos simultaneously. McNamara et al. (2025) achieved high-throughput patterning of Nodal signaling in zebrafish embryos, simultaneously testing the influence of illumination intensity and pattern geometry in embryos lacking endogenous Nodal signaling (Fig. 1D). Although the rescue was incomplete, the systematic testing of multiple input regimes will be important for extending these approaches to new developmental patterns.

## Challenges and progress at the frontiers of developmental optogenetics

Neuroscience and developmental biology share key features that make them ideal substrates for optogenetic studies: In both the brain and developing embryo, biochemical information is stored spatially and varies substantially over time. However, the application of optogenetic systems to development still faces engineering and practical challenges that limit broad application due to the different light-sensitive domains used and the characteristic length scales and timescales involved. In this section, we highlight the substantial progress toward addressing these challenges, recent innovations that make possible a wider use of optogenetics in developmental biology, and remaining technical barriers that should be the focus of future advances to further unlock the potential of these powerful techniques.

### Combining optogenetic stimulation and fluorescence imaging

**Figure 5.**
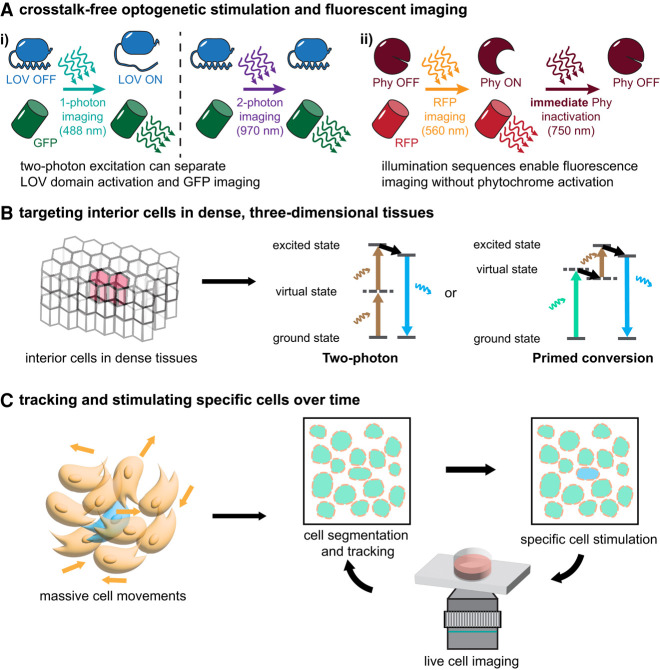
Challenges and progress in combining precise optogenetic stimulation and live imaging. (*A*) Two strategies for avoiding spectral cross-talk are to use two photon excitation to selectively image GFP without stimulating LOV domains (panel *i*) and to use phytochrome-based tools that can be inactivated by a pulse of inactivating light after imaging to limit downstream optogenetic signaling (panel *ii*). (*B*) Developing tissues are usually three-dimensional and dense, making it challenging to target specific cells in the interior region. Two photon and primed conversion techniques have been leveraged to achieve 3D confinement in dense tissues for optogenetic stimulation. (*C*) Cells move extensively during development, so consistently tracking and stimulating specific cells is difficult. Modern computational advances in deep learning-based methods and “smart microscopy” have enabled more precise and consistent optogenetic stimulation of target cells.

Fluorescence imaging is often essential for analyzing developmental dynamics, yet its integration with optogenetic perturbation poses several challenges. One major obstacle is spectral cross-talk: Optogenetic actuators and fluorescent reporters often share overlapping excitation or emission spectra (Fig. 5A). Many optogenetic tools, including flavin-utilizing LOV domains and cryptochromes, are excited by 400−500 nm blue light (Christie et al. 1999; Salomon et al. 2000; Zoltowski et al. 2007; Yazawa et al. 2009; Kennedy et al. 2010) and are typically activated at lower light intensities than those used for imaging. Therefore, detection of blue, cyan, or green fluorescent proteins typically causes unintended activation of these tools.

One strategy to circumvent spectral cross-talk is to use two photon excitation for imaging or photoactivation (Fig. 5A, panel i). Homans et al. (2018) discovered that LOV domains have significantly blue-shifted two photon absorption spectra and long fluorescence lifetimes compared with fluorescent proteins with similar single-photon spectra. Singh et al. (2022) took advantage of this shift in two photon excitation, showing that it is possible to perform two photon GFP/mCherry imaging in *Drosophila* embryos without unwanted activation of AsLOV2-based optogenetic tools that can instead be excited using standard single-photon blue-light illumination. Conversely, Kinjo et al. (2019) fused a blue fluorescent protein (BFP) to various optogenetic tools, showing that two photon stimulation of BFP could be used to activate the optogenetic tool by FRET-based energy transfer for optogenetic stimulation.

A second strategy to overcome spectral limitations and also enable deeper tissue penetration with reduced phototoxicity involves stimulating phytochrome-based optogenetic tools with more sophisticated illumination sequences (Fig. 5A, panel ii). Phytochromes are typically photochromic, switching between conformational states in response to two different wavelengths of light (e.g., PhyB, which is activated by 650 nm red light and inactivated by 740 nm near-infrared light). This bidirectional switching can be advantageous when combining imaging and optogenetic stimulation, because virtually any fluorophore can be imaged without cross-talk as long as imaging is followed by a pulse of near-infrared light to rapidly restore the phytochrome to its “off” state before appreciable optogenetic activation has occurred.

### Stimulating precise volumes in dense, three-dimensional tissues

Another challenge is the targeting of precise volumes in a tissue for optogenetic stimulation (Fig. 5B). Many embryos are dense, three-dimensional structures, and light scattering can complicate any optical technique. This challenge is even greater for optogenetic stimulation than for imaging, because in imaging, out-of-focus excitation light can be later rejected using techniques such as confocal pinholes, whereas for optogenetic tools, this light would directly lead to unintended activation. These challenges explain why virtually all studies incorporating developmental optogenetics have been carried out in model systems and developmental stages that can be patterned using a two-dimensional light stimulus (e.g., the *Drosophila* syncytial blastoderm and micropatterned mammalian embryo models).

How might precise three-dimensional stimulation be achieved? Two photon optogenetic stimulation is a particularly useful solution, as it permits activation in a precisely defined volume (Izquierdo et al. 2018). A second multiphoton approach, primed conversion, shows great promise but has not yet been applied to optogenetic stimulation. Unlike traditional two photon excitation, which relies on near-simultaneous absorption of two equal-energy photons to complete an electronic energy transition, primed conversion relies on single-photon absorption to populate an intermediate energy state, followed by absorption of a second photon for complete photoexcitation (Mohr and Pantazis 2018). Dempsey et al. (2015) reported primed conversion in the Dendra2 photoconvertible fluorescent protein, triggering a green-to-red change in fluorescence with a combination of 488 nm and NIR excitation. Restricting activation to a region of overlap between two relatively low-intensity illumination patterns makes primed conversion likely to be useful for optogenetic activation, but whether any optogenetic tools are primed-convertible is yet to be determined.

### Tracking and stimulating target cells during dynamic developmental processes

In the adult brain, cells can occupy static positions for decades, so optogenetics in neuroscience typically involves applying light patterns to a fixed field of cells. In contrast, developmental processes often include extensive cell movements that occur on the same timescales as the biochemical and mechanical signals that regulate them. A major challenge in developmental optogenetics thus involves identifying hundreds or thousands of cells of interest and tracking them in real time while updating the illumination inputs delivered to each cell (Fig. 5C). Fortunately, achieving this goal can benefit from advances in computational approaches, particularly deep learning-based methods, which have dramatically improved our ability to segment, track, and analyze cells (Stegmaier et al. 2016; Stringer et al. 2021; Wen et al. 2021; Soelistyo et al. 2023; Bragantini et al. 2025). There has been a recent flurry of work at the “smart microscopy” frontier, showing that it is feasible to perform real-time cell segmentation, tracking, and optogenetic stimulation on thousands of cells in a single experiment (Oatman et al. 2025; Passmore et al. 2025; Hinderline et al. 2026). We look forward to the extension of these approaches to developmental systems to enable stimulation of target cells even during processes like gastrulation, in which cell positions and fates are rapidly changing.

### Patterning multiple outputs

**Figure 6.**
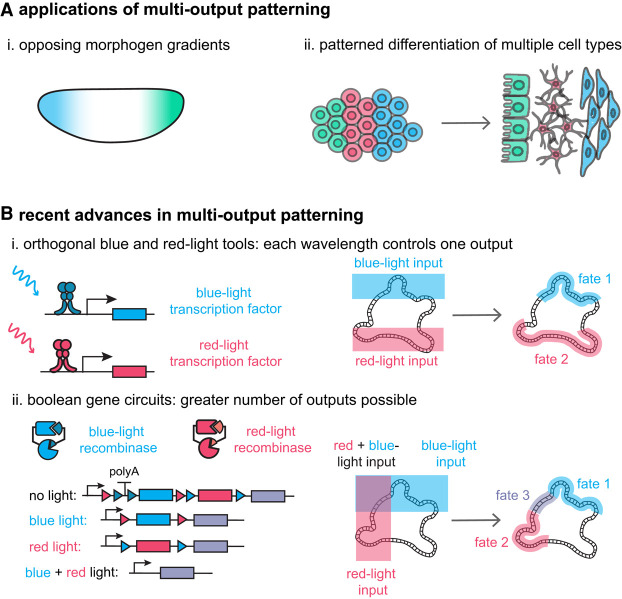
Scenarios in which multioutput optogenetic patterning is needed and frontiers of multioutput patterning. (*A*). Many developmental processes require the coordinated regulation of multiple components. Opposing morphogen gradients (panel *i*) and patterned differentiation of multiple cell types (panel *ii*) are two common examples in which at least two signaling pathways or genes are involved. (*B*) Current approaches for multioutput patterning include multichromatic control of transcription or signaling pathways with two orthogonal optogenetic tools (panel *i*) and multiplexed transcription control with two orthogonal light-induced recombinases coupled with Boolean gene circuits (panel *ii*). The approach shown in panel *ii* enables the number of outputs patterned to exceed the number of inputs.

Developmental processes are inherently complex, often requiring the coordinated regulation of multiple signaling pathways and gene networks. For instance, *Drosophila* anterior–posterior axis formation requires the opposing morphogen gradients of *bicoid* and *nanos*, and vertebrate neural tube patterning needs the antiparallel gradients of Sonic hedgehog signaling emanating from the ventral pole and Wnt and bone morphogenetic protein (BMP) signaling from the dorsal pole (Fig. 6A, panel i; Frohnhöfer and Nüsslein-Volhard 1986; Jessell 2000; Briscoe and Small 2015; Zagorski et al. 2017; Vetter and Iber 2022). The latter case is also an example where multiple cell types (e.g., motor neurons and ventral neurons) need to be patterned at the correct positions for the tissue morphogenesis (Fig. 6A, panel ii; Briscoe and Ericson 2001; Dessaud et al. 2008; Le Dréau and Martí 2012). Manipulating a single output is often insufficient to fully recapitulate or interrogate these embryogenic processes. The experimentalist may also wish to control multiple sequential processes, such as successive expression of identity-determining transcription factors in the same cell lineage to recapitulate progressive differentiation in development. As a result, there is a growing demand for multiplexed optogenetic control, where light inputs control different biochemical events in the same specimen.

Perhaps the simplest approach for multiplexing is to develop optogenetic actuators with nonoverlapping excitation spectra. Multicolor control in mammalian cells was demonstrated as early as 2013 using UVB-inducible, blue-light-inducible, and red-light-inducible gene switches (Müller et al. 2013). However, due to the spectral overlap among photosensory proteins, this system afforded only two orthogonal channels: pairing of the red-light system with either the UVB or blue-light system (Fig. 6B, panel i). Kramer et al. (2021) used a similar strategy to achieve orthogonal control of two signaling pathways: the RAF/ERK and AKT signaling pathways. The development of new photochromic optogenetic tools with different spectral properties may enable more sophisticated sequences of activating and inactivating wavelengths to address three or more proteins in future studies (Jang et al. 2023).

A second approach involves constructing synthetic gene circuits to widen the range of outputs that can be triggered by a limited number of stimuli. Using binary encoding, two inputs could drive up to four distinct output states (e.g., dark, blue only, red only, and blue + red) (Fig. 6B, panel ii). In an important recent study, Tous et al. (2025) introduced Boolean logic gates downstream from blue-light-controlled and red-light-controlled recombinases to achieve multiplexed patterning through DNA excision. They coupled a red-light-inducible Flp recombinase with a blue-light-inducible Cre recombinase and arranged three fluorescent proteins in tandem as reporters. An important constraint of this system is that the control achieved via this system is binary and irreversible, which prevents the induction of graded or pulsatile activity states but could be advantageous in cases where permanent gene expression is desired. Future developments are required to develop optogenetic toolkits with multioutput, dynamic, and reversible control.

### Extending optogenetics to mammalian developmental systems

How can we extend the reach of optogenetics to any developmental system of interest? At a glance, it would appear that there is no limitation, because the biochemistry of light-based proteins should operate similarly regardless of cellular context. This simple view belies a surprising degree of complexity in extending optogenetic tools across diverse model systems. While optogenetics has been broadly applied to studying *Drosophila* and zebrafish embryogenesis, its application has been much more limited in many other contexts, particularly in studies of mammalian development.

The challenges of extending optogenetics to mammalian developmental systems mainly lie in two aspects. First, the light penetration challenges discussed above are taken to the extreme in mammals, limited by the three-dimensional architecture of the embryo and further constrained by the intrauterine environment of mammalian development. Second, genetic modification is inherently more demanding in mammalian systems due to the challenges of site-specific genomic integration and transgene silencing.

As for the first challenge, mammalian embryos and embryo-like models can be large and dense (by gastrulation, a mouse embryo or gastruloid contains ∼10^5^ cells), making both imaging and optical stimulation challenging. The intrauterine development of mammalian embryos also makes them less accessible to precise light stimulation. Blue light penetrates tissue relatively poorly at depths above ∼1 mm, further complicating the use of most optogenetic tools (Clement et al. 2005; Ash et al. 2017; Senova et al. 2017). Red light, with a tissue penetration depth of ∼6 mm, may be a more suitable candidate for in vivo mammalian applications (Clement et al. 2005).

Nevertheless, despite these obstacles, researchers have proved the feasibility of in vivo and ex vivo light stimulation in mammalian embryogenesis. Five years after the development of a photoactivatable split Cas9 (paCas9) tool based on blue-light-induced dimerization of the VVD photoreceptor (Nihongaki et al. 2015), Takao et al. (2020) successfully used paCas9 to disrupt the leukemia inhibitory factor (LIF) gene in mouse uteri and block embryo implantation after external illumination of the entire animal with a 1.5 W/m^2^, 450 nm blue LED light source. It is likely that in this system, the inefficiency of blue-light penetration is offset by the long integration time of the VVD photoreceptor and the permanent genomic modification triggered by paCas9, enabling external illumination to trigger a potent response. In this implantation system, paCas9 offers great potential in elucidating the spatiotemporal role of implantation-associated molecules and providing a therapeutic strategy for temporal control of reproductive functions in vivo. Other than stimulating the entire animal, light has also been applied to early mouse preimplantation embryos in vitro. Morikawa et al. (2020) developed a light-induced split Cre recombinase and demonstrated that it could be used for light-induced Cre recombination in early mouse embryos. They collected fertilized eggs and illuminated them with blue light at E1.5–E2.5 and E2.5–E3.5, yielding successful cell labeling in homozygous mouse embryos. This new optogenetic recombination system provides a powerful tool for lineage tracing in early mouse embryos.

The second challenge is genetic introduction of the tools themselves. Many optogenetic tools rely on the association between multiple proteins, requiring the introduction of large multicistronic cassettes. One difficulty that is particularly evident in mammalian embryos and stem cell models is the robust silencing of transgenes that can occur during cell differentiation (Cabrera et al. 2022). Even though stable gene expression can be achieved in undifferentiated pluripotent stem cells, the expression is often lost upon differentiation due to epigenetic (DNA methylation and histone modification) silencing (Cabrera et al. 2022). One solution to this problem may involve adopting combinations of “tricks” for retaining expression: ubiquitous chromatin opening elements (UCOEs) to prevent silencing (Williams et al. 2005; Zhang et al. 2007, 2010; Müller-Kuller et al. 2015; Yanagi et al. 2025), introns such as those found in the CAG promoter system to mimic the splicing found in endogenous genes (Ramezani et al. 2000; Uenaka et al. 2026), and targeting to safe harbor loci or essential genes to promote sustained expression. It is also incompletely known how silencing depends on properties of the transgene sequence, a problem that would be well suited to high-throughput experimental and deep learning computational approaches. Advances in transgene antisilencing strategies would be expected to substantially enhance the reliability and applicability of optogenetic tools in developmental systems.

Despite the challenges associated with applying optogenetics in mammalian systems, researchers have circumvented several limitations by using isolated cells/tissues from mice or stem cell-derived organoids and answered significant questions in development. For instance, a series of studies from the Kageyama group (Imayoshi et al. 2013; Yoshioka-Kobayashi et al. 2020; Isomura et al. 2017, 2026) applied GAVPO in mammalian cell cultures and organoids to dissect how oscillatory expression of transcription factors stabilizes progenitor states and to elucidate the mechanisms underlying synchronized oscillations in the segmentation clock. Stem cells and stem cell-derived models thus provide a powerful platform to alleviate some obstacles in mammalian optogenetics, such as generating transgenic mice and delivering light to intrauterine embryos, while fulfilling the great potential of optogenetics.

It is exciting to imagine where developmental optogenetics will go from here. Sophisticated measurements have revealed single-cell lineages, gene expression states, and signaling activity using a combination of imaging, next-generation sequencing, and biosensor technologies, providing a detailed map of natural developmental processes that is ripe for perturbation. Optogenetic tool development is proceeding at an ever-accelerating pace, with tools now available to manipulate cells’ signaling states, gene expression, and even genomic sequence with high spatiotemporal precision and low leakiness. At the same time, techniques are advancing rapidly for spatially precise optical stimulation and delivery of synthetic genetic cargo. It has never been a better time to be a quantitative developmental biologist.
